# Supplementary material for: Cellular and biophysical barriers to lipid nanoparticle mediated delivery of RNA to the cytosol
Source: Nat Commun. 2025 Jul 1;16:5354. doi: 10.1038/s41467-025-60959-z (PMC12217163; doi:10.1038/s41467-025-60959-z)
Supplement: Supplementary file 1 — Supplementary Information [file 41467_2025_60959_MOESM1_ESM.pdf]

Supplementary Information for

Cellular and biophysical barriers to lipid nanoparticle mediated delivery of RNA to the cytosol

Johanna M. Johansson*, Hampus Du Rietz*, Hampus Hedlund*, Hanna C. Eriksson, Erik Oude Blenke, Aditya Pote, Said Harun, Pontus Nordenfelt, Lennart Lindfors & Anders Wittrup§

*Equal contribution.
§Corresponding author. Email: anders.wittrup@med.lu.se

This PDF-file includes:
Supplementary Figures 1–10
Supplementary Note 1

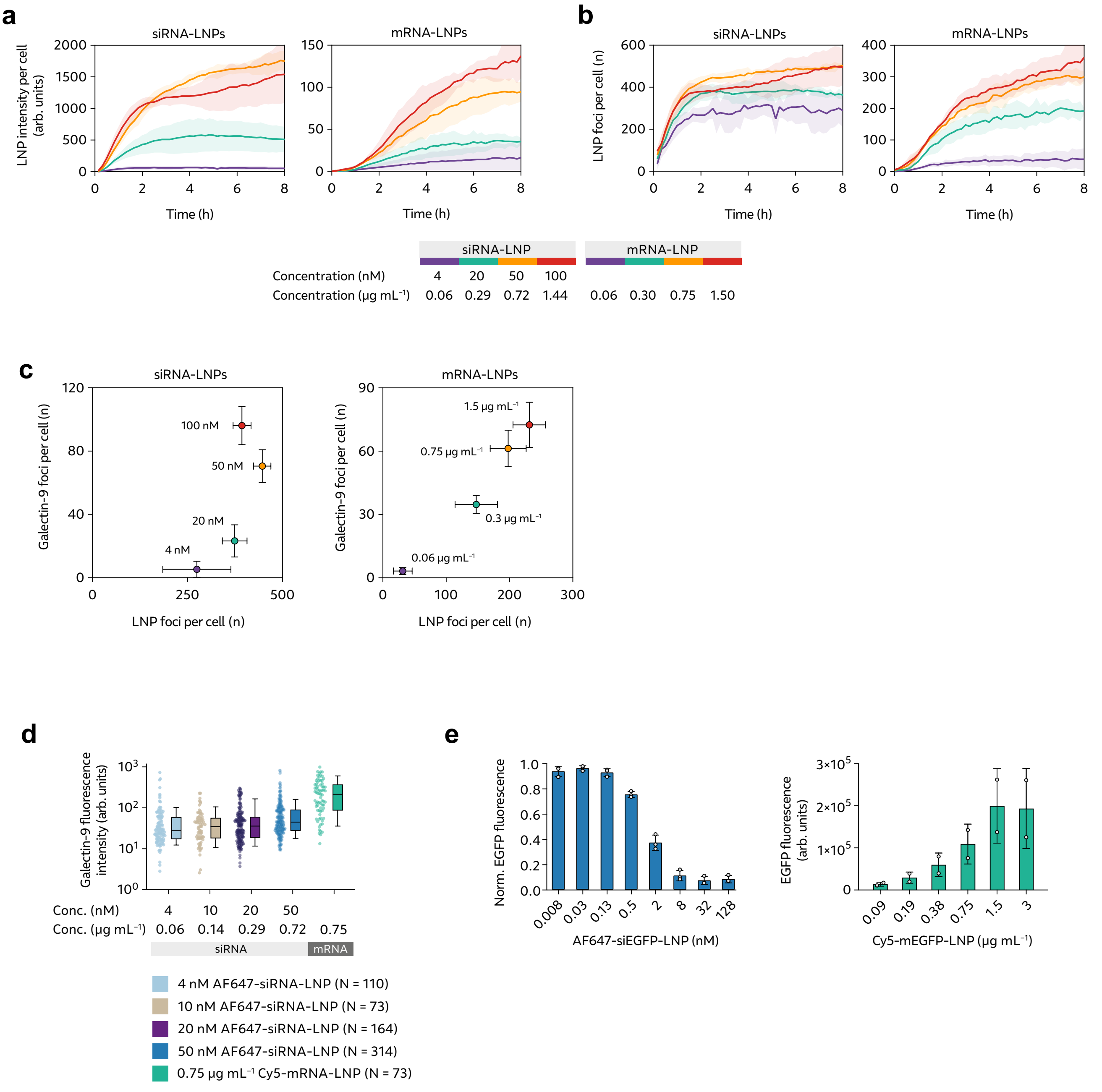

Supplementary Fig. 1 | Characterizing endosomal release of RNA- LNPs. HeLa-Gal9-YFP cells were incubated with fluorescently labeled siRNA- or mRNA-LNPs during live-cell widefield microscopy. **a** Images were acquired every 10 min for 8 h during LNP incubation, quantifying the the AF647/Cy5 fluorescence intensity in a typical cell or (**b**) the number of LNP foci per cell. Line is mean, shade is s.d. N = 4 technical replicates (cells = 101, 105, 117, 106 and 124, 110, 120, 102) from 2 independent experiments for each RNA modality, with 4, 20, 50, 100 nM siRNA- and 0.06, 0.30, 0.75, 1.50 µg mL⁻¹ mRNA-LNPs, respectively. **c** Number of galectin-9 positive vesicles and the number of LNPs in a typical cell after 3 hours of incubation with indicated doses. Mean ± s.d. from Fig. 1a and Supplementary Fig. 1b. N = 4 technical replicates from 2 independent experiments. **d** Galectin-9 fluorescence intensity measured between ~44–75 s after start of recruitment. Circles are pooled individual vesicle from at least 2 independent experiments. Boxes are median ± i.q.r; whiskers are 10–90 percentile. **e** HeLa cells stably expressing d1-EGFP (left) or wild-type (right) were incubated with AF647-labeled siRNA-LNPs or Cy5-labeled mRNA-LNPs, targeting or encoding EGFP (siEGFP; mEGFP), respectively, at the indicated concentrations. EGFP fluorescence intensity was evaluated using flow cytometry. Bars show mean ± s.d. of 3 (siRNA) or 2 (mRNA) independent experiments (circles).

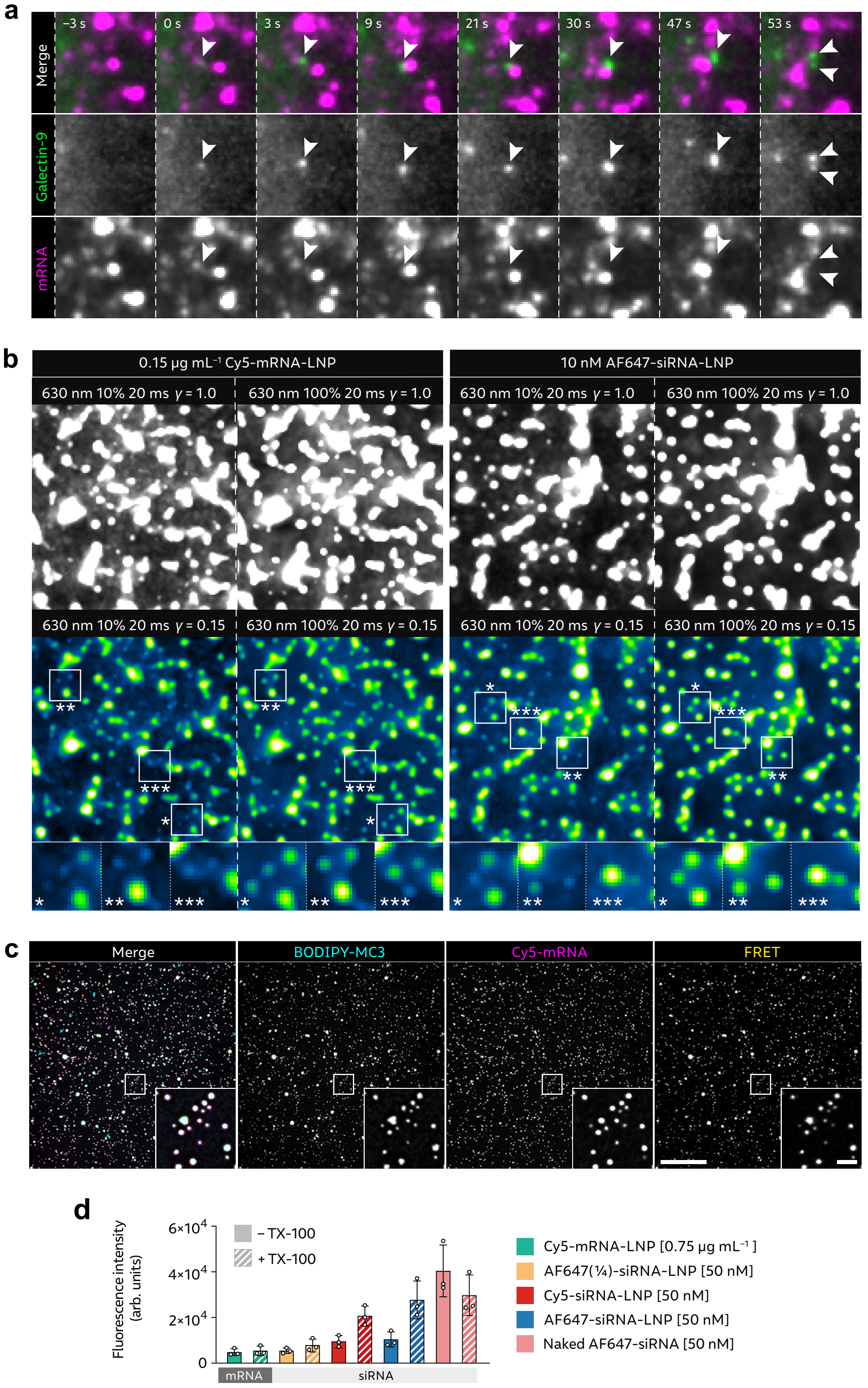

Supplementary Fig. 2 | Detection of RNA-LNPs. a HeLa-Gal9-YFP cells were incubated with 0.75 µg mL⁻¹ Cy5-mRNA-LNPs while acquiring images with a widefield microscope. Arrowheads indicate galectin-9 recruitment to a structure with no detectable mRNA content. Images are representative of 796 damage events from 3 independent experiments. **b** Samples with mRNA-LNP (left) or siRNA-LNP (right) were prepared in diH2O on glass slides and images using standard experimental acquisition parameters (left subpanels) or sensitivity optimized parameters (right subpanels). Brightness and contrast were adjusted separately for all images. Gamma was adjusted in pseudocolored images, to aid comparison of individual LNPs between image sets. **c** 0.15 µg mL⁻¹ Cy5-mRNA MC3-BODIPY LNPs were prepared in diH2O in a glass slide and imaged using a VT-iSIM microscope. Images are representative of two independent experiments. Scale bar, 20 µm; details, 2 µm. **d** Naked AF647-siRNA, siRNA-LNP labeled with AF647, AF647¼ or Cy5 (all 50 nM) and Cy5-mRNA-LNP (0.75 µg mL⁻¹) were prepared in OptiMEM. Triton-X-100 (TX-100, final concentration 1 %) was added to disrupt LNPs. AF647/Cy5 fluorescence was measured with a spectrophotometer. Bars show mean ± s.d. of 3 independent experiments (circles).

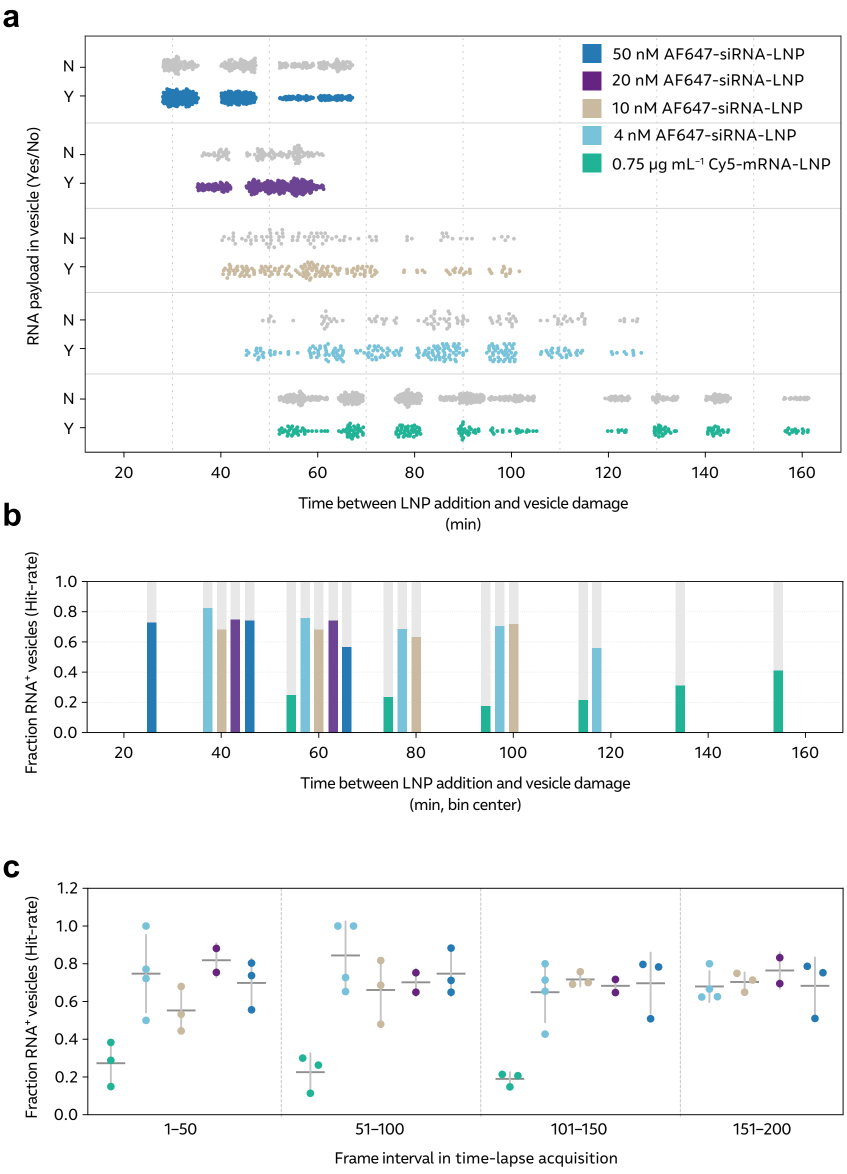

Supplementary Fig. 3 | Hit rate of RNA payload in damaged vesicles. HeLa-Gal9-YFP cells were incubated with 4–50 nM siRNA-LNPs or 0.75 µg mL^{-1} mRNA-LNPs as indicated and imaged using fast live-cell microscopy. Events with de novo recruitment of galectin-9 were identified and evaluated with respect to presence or absence of detecteble RNA payload in the galectin-9^{+} structure. **a** All identified damage events are shown with RNA^{+} and RNA^{-} events separated (colored and gray points, respectively) for the indicated conditions, plotted according to the corresponding time of the damage event in relation to start of continuous LNP incubation. Broken vertical gray lines indicate bins used in b, and horizontal gray lines separate the different conditions. **b** The fraction of all damaged vesicles with detectable payload were calculated per condition after binning data. Center of bins are indicated, bin width is 20 min. Colored and gray bars show RNA^{+} and RNA^{-} vesicles, respectively. **c** Fraction of all damaged vesicles (per condition) with detectable RNA payload, presented separately for three (mRNA) or four (siRNA) intervals during image acquisition. Acquisitions were typically 150 or 200 frames (time-points), for mRNA- and siRNA-LNPs, respectively. Lines are mean ± s.d. N = independent experiments. Data in **a–c** is from 4, 3, 2, 3 and 3 independent experiments (number of vesicles (Nv) and number of cells (Nc) shown in parenthesis (Nv;Nc).= (337;208), (255;117), (484;116), (1019;135) and (952;205), with 4, 10, 20, 50 nM siRNA- and 0.75 µg mL^{-1} mRNA-LNPs, respectively.

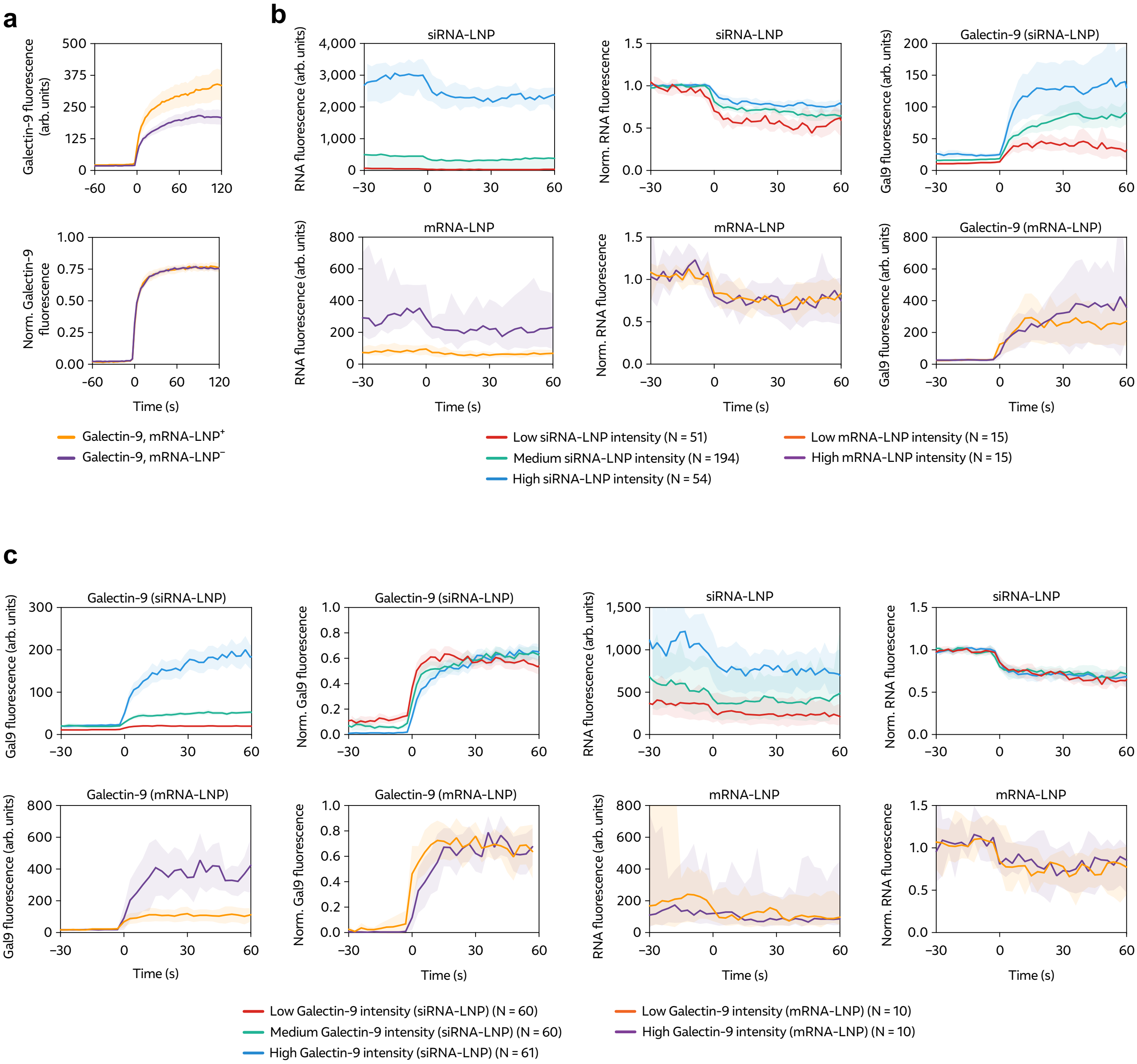

Supplementary Fig. 4 | Sub-group analysis of RNA-LNP release kinetics. HeLa-Gal9-YFP cells were incubated with 4–50 nM siRNA-LNPs or 0.75 µg mL^{-1} mRNA-LNPs as indicated and imaged using fast live-cell microscopy. **a** Galectin-9 recruitment to endosomes with or without detectable mRNA payload (mRNA^{+} or mRNA^{-}, respectively). **b**, **c** Single vesicle measurements of damaged endosomes containing siRNA-LNPs (top) or mRNA-LNPs (bottom) were sub-grouped based on the mean (**b**), pre-damage RNA intensity, or (**c**), galectin-9 intensity ~45–75 s after start of recruitment, into two (mRNA) or three (siRNA) groups. Data is shown as raw values (with background correction) or normalized to the mean vesicle RNA intensity before damage or min and max galectin-9 intensity per vesicle. Lines are median or mean, shades are 95% CI of median or mean, for RNA-LNP and galectin-9, respectively.

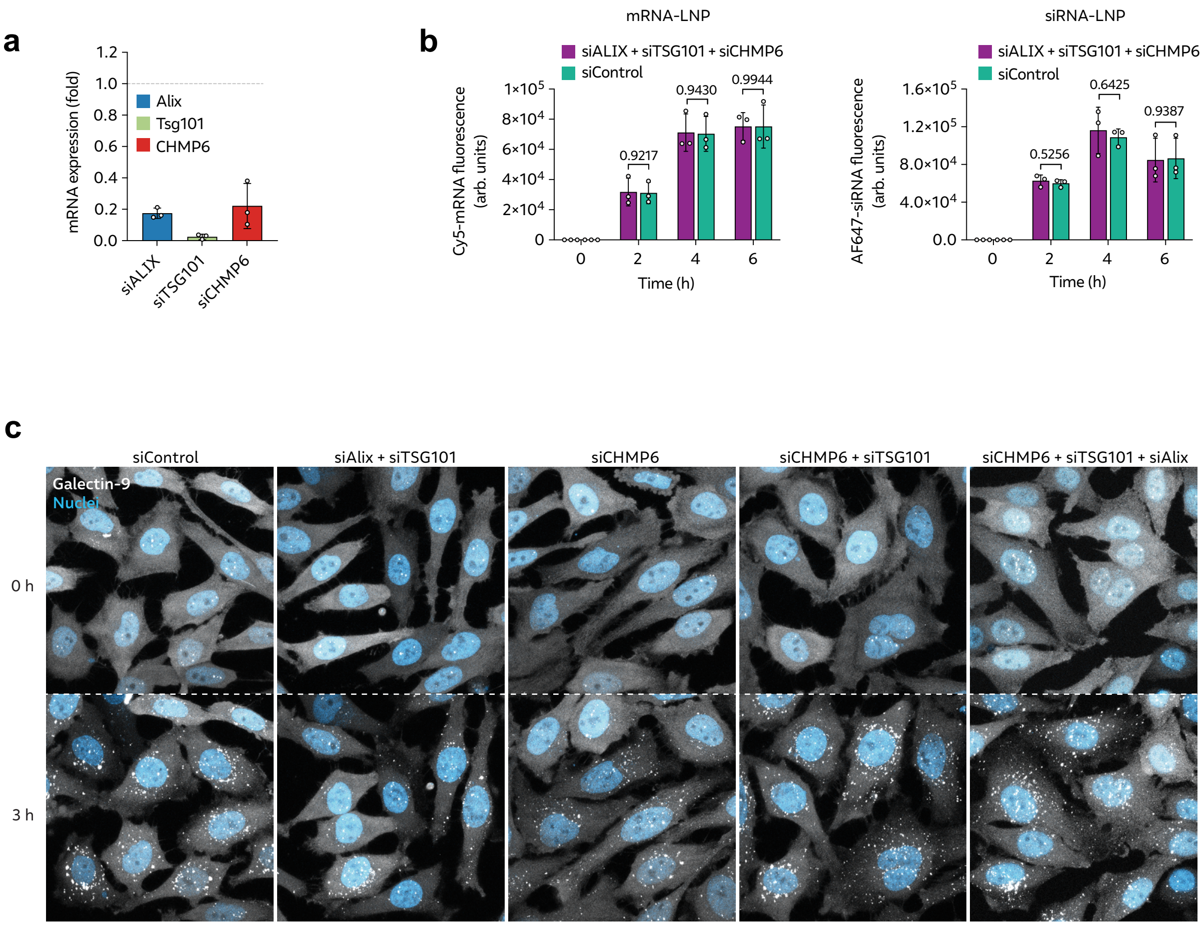

Supplementary Fig. 5 | ESCRT knockdown does not affect LNP internalization. HeLa-Gal9-YFP cells were transfected with siRNA targeting Alix, Tsg110 and CHMP6 or negative control siRNA. Experiments were performed 24 h after transfection. **a** Target mRNA knockdown evaluated using RT-qPCR ($\Delta\Delta C_T$). Mean ± s.d. from three independent experiments. **b** Cells were incubated with 0.5 µg mL^{-1} Cy5-mRNA or 20 nM AF647-siRNA LNPs for the times indicated. Fluorescence intensity of internalized RNA was then determined by flow cytometry. Mean ± s.d. from three independent experiments. Unpaired two-sided Student's t-test. **c** HeLa-Gal9-YFP cells were continuously incubated with 0.5 µg mL^{-1} mRNA-LNPs after transfection with ESCRT-targeting siRNA (combinations detailed in the plots). The galectin-9 response was monitored using confocal microscopy. Images are representative of 3 independent experiments.

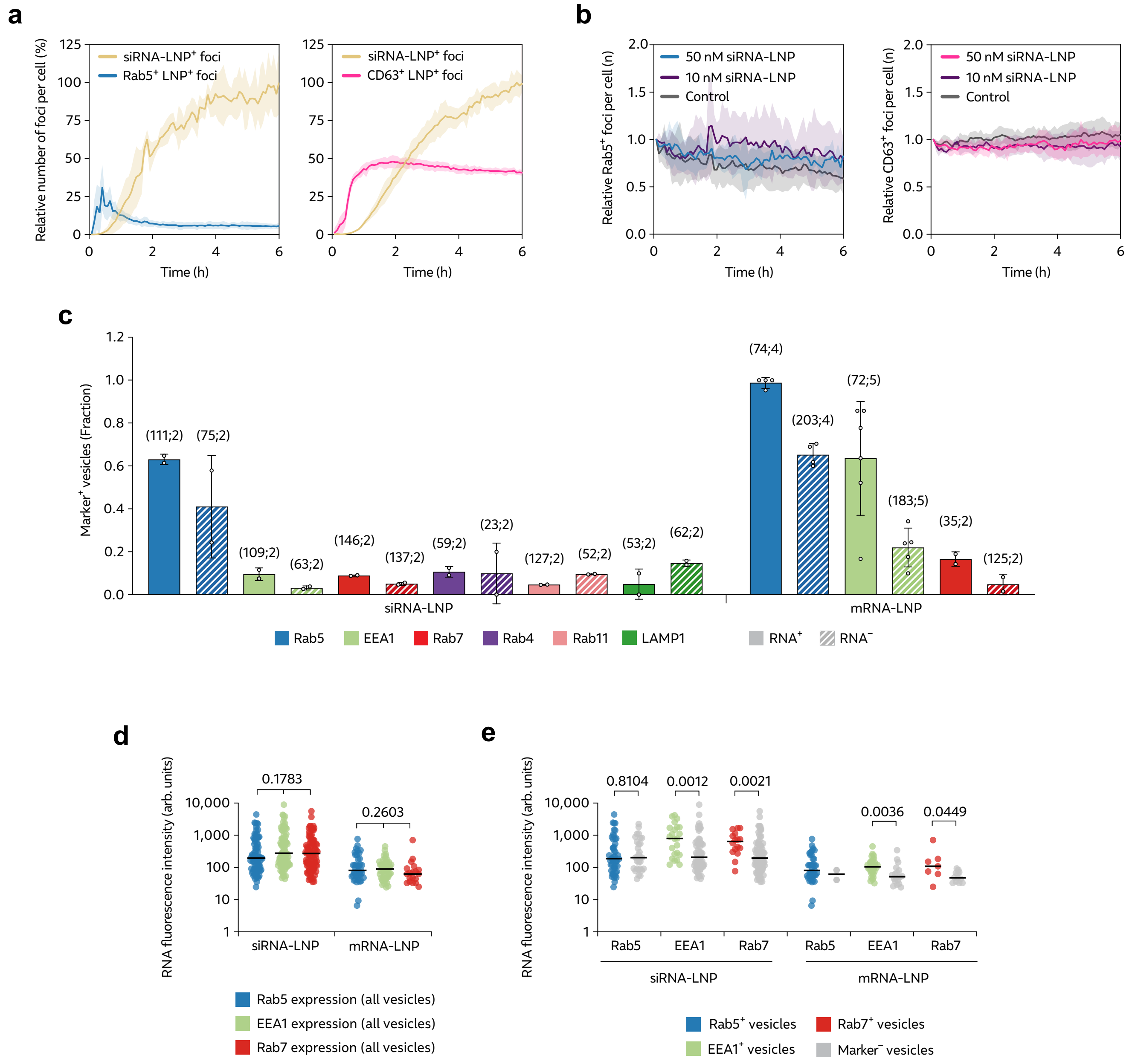

Supplementary Fig. 6 | Distribution of LNPs and classification of damaged endosomal compartment identity. a, b HeLa cells expressing GFP-Rab5 or GFP-CD63 were incubated with fluorescently labeled siRNA-LNPs during live-cell confocal microscopy, acquiring images every 5 min for 8 h. **a** Fraction of siRNA-LNP foci colocalizing with indicated compartment marker (left) and the uptake of siRNA-LNPs (right) in a typical cell over time, during 50 nM siRNA-LNP incubation. siRNA-LNP uptake was normalized to the largest mean in the data set. Line is mean, shade is s.d. N = 6 technical replicates as shown in (**b**). **b** Number of foci per cell for the indicated compartment marker during incubation with specified siRNA-LNP doses, normalized to start of image acquisition. Line is mean, shade is s.d. N = 6 technical replicates, with Rab5-expressing cells (102, 85, and 98 cells for untreated, 10 nM, and 50 nM siRNA-LNP treatment, respectively) and CD63-expressing cells (250, 227, and 232 cells for untreated, 10 nM, and 50 nM siRNA-LNP treatment, respectively). Data is from two independent experiments per compartment marker. **c–e** HeLa-Gal9-YFP cells were incubated with 4–50 nM siRNA-LNPs or 0.75 µg mL^{-1} mRNA-LNPs as indicated and imaged using fast live-cell microscopy. **c** Fraction of all damaged vesicles (per condition) with presence of the indicated compartment marker (observer dependent classification), presented separately for RNA$^+$ and RNA$^-$ endosomes (filled and striped colors, respectively). Bars show mean ± s.d. of at least 2 independent experiments (circles) per condition. Number of events (N_E) and experiments (N_X) are shown in parentheses as (N_E;N_X). Number of analyzed cells (siRNA-, mRNA-LNPs respectively): Rab5: 79, 138; EEA1: 96, 143; Rab7: 128, 85; Rab4: 27; Rab11: 63; LAMP1: 51. **d** RNA fluorescence intensity of all analyzed LNP$^+$ vesicles in cells expressing the indicated compartment marker, determined before galectin-9 recruitment. Statistics: Kruskall-Wallis test. **e** RNA fluorescence intensity of marker$^+$ (color) and marker$^-$ (gray) vesicles with indicated compartment marker. Statistics: Mann-Whitney test. N = analyzed events, numbers provided in Figure 3c.

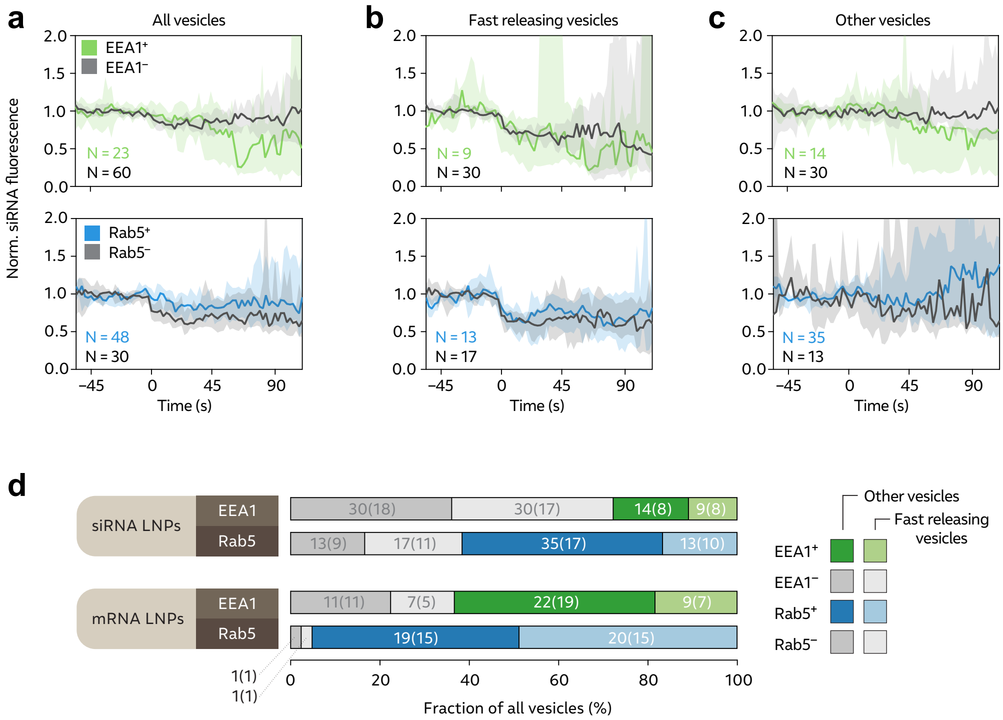

Supplementary Fig. 7 | LNP mediated endosomal release of RNA in early endosomal compartments. HeLa-Gal9-YFP cells expressing Rab5- or EEA1-mScarlet were incubated with 50 nM AlexaFluor647-labelled siRNA-LNPs, or 0.75 µg mL[a] Cy5-labeled mRNA-LNPs. Cells were monitored with high-speed live-cell widefield microscopy, and de novo formation of galectin-9 foci were identified. LNP$^+$ endosomes showing galectin-9 recruitment were tracked in 4D, measuring the endosomal marker fluorescence intensity. A dynamic intensity threshold (0.5 × 90th percentile intensity per cell) was applied to determine the fraction of vesicles with presence of the respective markers at a ~10 s interval centered around start of galectin-9 recruitment. Traces were aligned in time so that t = 0 is the time-point with first detectable galectin response. RNA intensity values were normalized to the mean vesicle intensity before damage. **a** All analyzed vesicles with siRNA-LNP payload. **b, c** Traces were subgrouped to separate release events showing (**b**) fast initial RNA release around t = 0, from (**c**) the remaining events. Lines are median, shades are 95% CI of median. N = vesicles. **d** Bars show fraction of fast releasing and other vesicles for vesicles that are marked by EEA1 or Rab5 (EEA1$^+$, Rab5$^+$), or are marker negative (EEA1$^-$, Rab5$^-$), for mRNA-LNPs and siRNA-LNPs respectively. Number of events (N_E) and cells (N_C) are shown as $N_E(N_C)$ for all conditions. Data is from at least two independent experiments per condition.

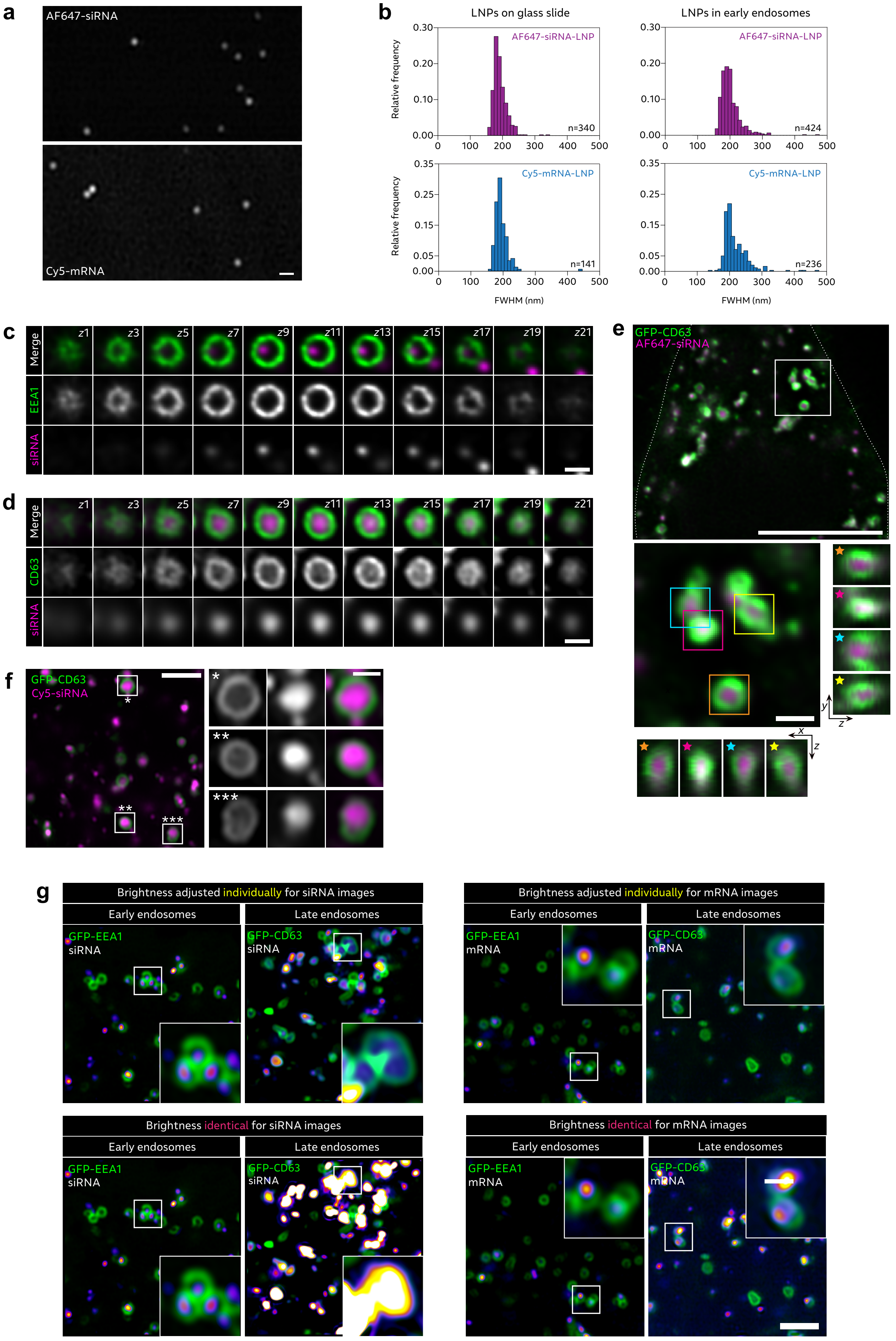
a
AF647-siRNA
Cy5-mRNA
b
LNPs on glass slide
LNPs in early endosomes
Relative frequency
AF647-siRNA-LNP
n=340
n=424
Cy5-mRNA-LNP
n=141
n=236
FWHM (nm)
c
Merge
EEA1
siRNA
z1
z3
z5
z7
z9
z11
z13
z15
z17
z19
z21
d
CD63
e
GFP-CD63
AF647-siRNA
f
Cy5-siRNA
g
Brightness adjusted individually for siRNA images
Brightness adjusted individually for mRNA images
Early endosomes
Late endosomes
GFP-EEA1
siRNA
mRNA
Brightness identical for siRNA images
Brightness identical for mRNA images

Supplementary Fig. 8 | Visualizing LNP disintegration with super-resolution microscopy. a,b AF647-siRNA-LNPs (20 nM) or Cy5-mRNA-LNPs (0.3 µg mL^{-1}) were diluted in diH2O in microscopy chamber slides and imaged using Airyscan confocal microscopy. Scale bar is 500 nm. **b** Full-width at half maximum (FWHM) of LNP-RNA, either in microscope slides or in $EEA1^{+}$ endosomes in HeLa cells expressing GFP-EEA1, incubated with LNP-siRNA-AF647 (100 nM) or LNP-mRNA-Cy5 (1.5 µg mL^{-1}) for 15–75 min. N = quantified LNP foci, from 10 experiments (AF647-siRNA-LNPs on glass) or 3 experiments (Cy5-mRNA-LNPs on glass, AF647-siRNA-LNPs and Cy5-mRNA-LNPs in early endosomes). **c–e** HeLa cells expressing GFP-EEA1 or GFP-CD63 were incubated with 100 nM AF647-siRNA LNPs for 30–90 min or 90–130 min for EEA1 or CD63 cells respectively. Z-stacks, with a 100 nm step-size, were acquired with VT-iSIM. Images are representative of 2 independent experiments. **c**, **d** scale bars are 500 nm. **e** orthogonal views were created with Fiji. White outlines indicate cell border. Scale bar, 10 µm; detail, 1 µm. **f** HeLa cells expressing GFP-CD63 were treated with Cy5-siRNA-LNPs (100 nM) for 45–120 min and imaged using Airyscan confocal microscopy. Images are representative of two experiments. g, HeLa cells expressing GFP-EEA1 or GFP-CD63 were treated with LNP-siRNA-AF647 (100 nM) or LNP-mRNA-Cy5 (1.5 µg mL^{-1}) for 15–75 min (EEA1) or 45–120 min (CD63) and imaged using Airyscan confocal microscopy. RNA channel brightness and contrast (LUT) were adjusted separately for top images (to highlight intra-luminal LNP appearance) and kept identical for bottom images. Brightness and contrast of EEA1 and CD63 images are kept constant. Images are representative of four (mRNA vs. CD63) or three (siRNA vs. EEA1, mRNA vs. EEA1 and siRNA vs. CD63) independent experiments. **f**, **g** Scale bar, 2 µm; detail, 500 nm.

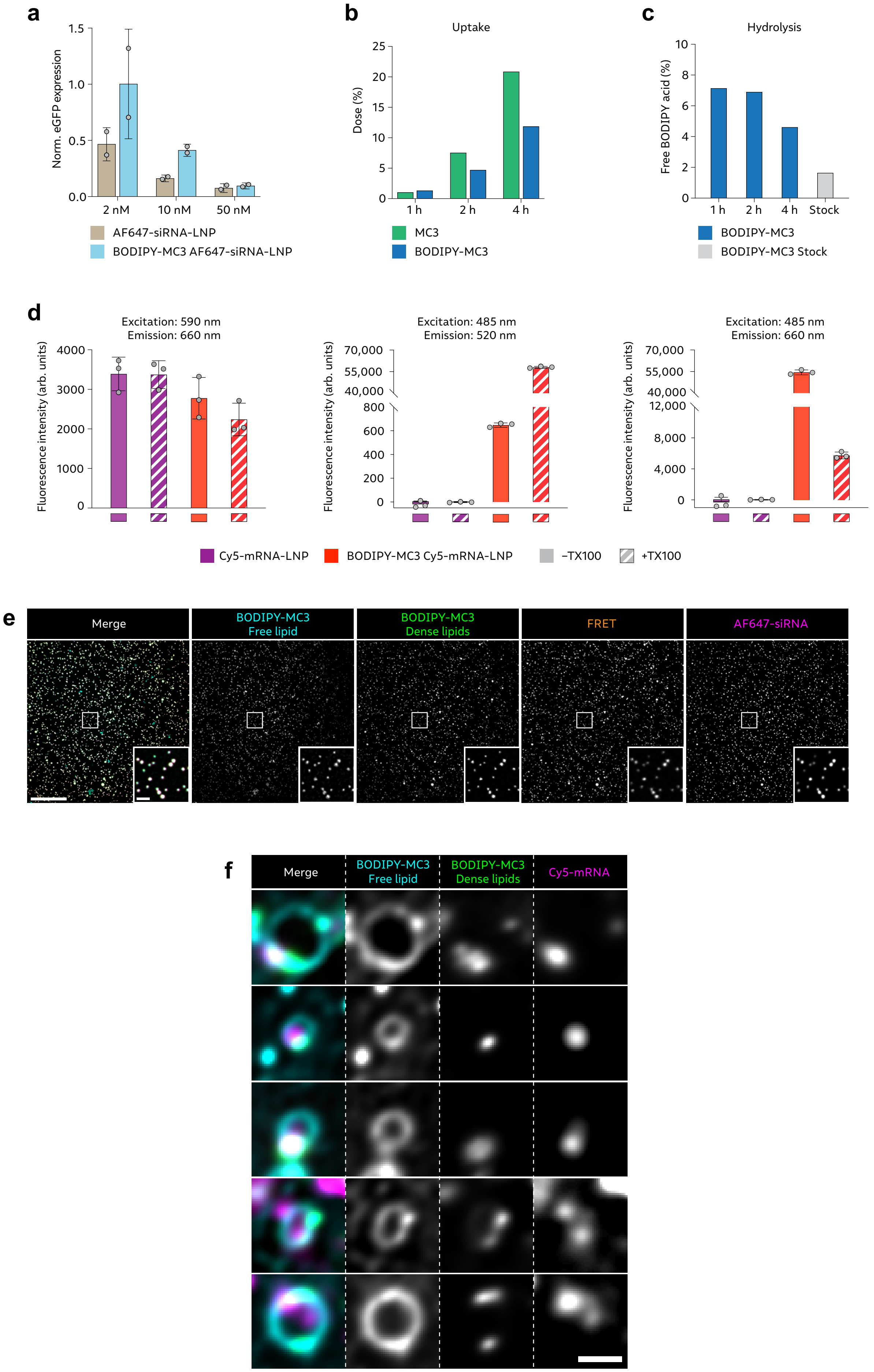
a
Norm. eGFP expression
1.5
1.0
0.5
0.0
2 nM
10 nM
50 nM
AF647-siRNA-LNP
BODIPY-MC3 AF647-siRNA-LNP
b
Uptake
Dose (%)
25
20
15
10
5
0
1 h
2 h
4 h
MC3
BODIPY-MC3
c
Hydrolysis
Free BODIPY acid (%)
10
8
6
4
2
0
1 h
2 h
4 h
Stock
BODIPY-MC3
BODIPY-MC3 Stock
d
Excitation: 590 nm
Emission: 660 nm
Fluorescence intensity (arb. units)
4000
3000
2000
1000
0
Excitation: 485 nm
Emission: 520 nm
70,000
55,000
40,000
800
600
400
200
0
Excitation: 485 nm
Emission: 660 nm
70,000
55,000
40,000
12,000
8,000
4,000
0
Cy5-mRNA-LNP
BODIPY-MC3 Cy5-mRNA-LNP
−TX100
+TX100
e
Merge
BODIPY-MC3
Free lipid
BODIPY-MC3
Dense lipids
FRET
AF647-siRNA
f
Merge
BODIPY-MC3
Free lipid
BODIPY-MC3
Dense lipids
Cy5-mRNA

Supplementary Fig. 9 | BODIPY-MC3 LNPs are functional and non-hydrolysed following internalization. a HeLa-d1-eGFP cells were incubated with AF647-siRNA or AF647-siRNA BODIPY-MC3 LNPs for 24 h and the eGFP knockdown was measured with RT-qPCR. Bars show mean ± s.d of 2 independent experiments (circles). **b**, **c** HeLa wild-type cells were incubated with 100 nM AF647-siRNA or AF647-siRNA BODIPY-MC3 LNPs for 1, 2 or 4 h. Cells were snap frozen followed by UHPLC-TOF-MS analysis. **b** Fraction of the total LNP dose (MC3) per well internalized by cells. **c** Fraction of total BODIPY acid not bound to MC3 (free BODIPY). N = 1 experiment. **d** Fluorescence intensity of Cy5-mRNA-LNPs and Cy5-mRNA BODIPY-MC3 LNPs (both 0.75 µg mL^{-1}) at the indicated excitation/emission wavelengths, measured using a spectrophotometer. To disrupt the LNPs, Triton-X-100 (TX-100, 1% final concentration) was added. Bars show mean ± s.d of 3 independent experiments (circles). **e** AF647-siRNA BODIPY-MC3 LNPs (10 nM) were prepared in diH2O in glass slides and imaged using standard experimental setting with VT-iSIM. Images are representative of 3 independent experiments. Scalebar, 20 µm; detail, 2 µm. **f** HeLa wildtype cells were treated with 1.5 µg mL^{-1} Cy5-mRNA BODIPY-MC3 LNPs for approximately 1–4 h and imaged using VT-iSIM. Brightness and contrast were adjusted separately for all images. Scale bar is 500 nm. Images are representative of 2 independent experiments.

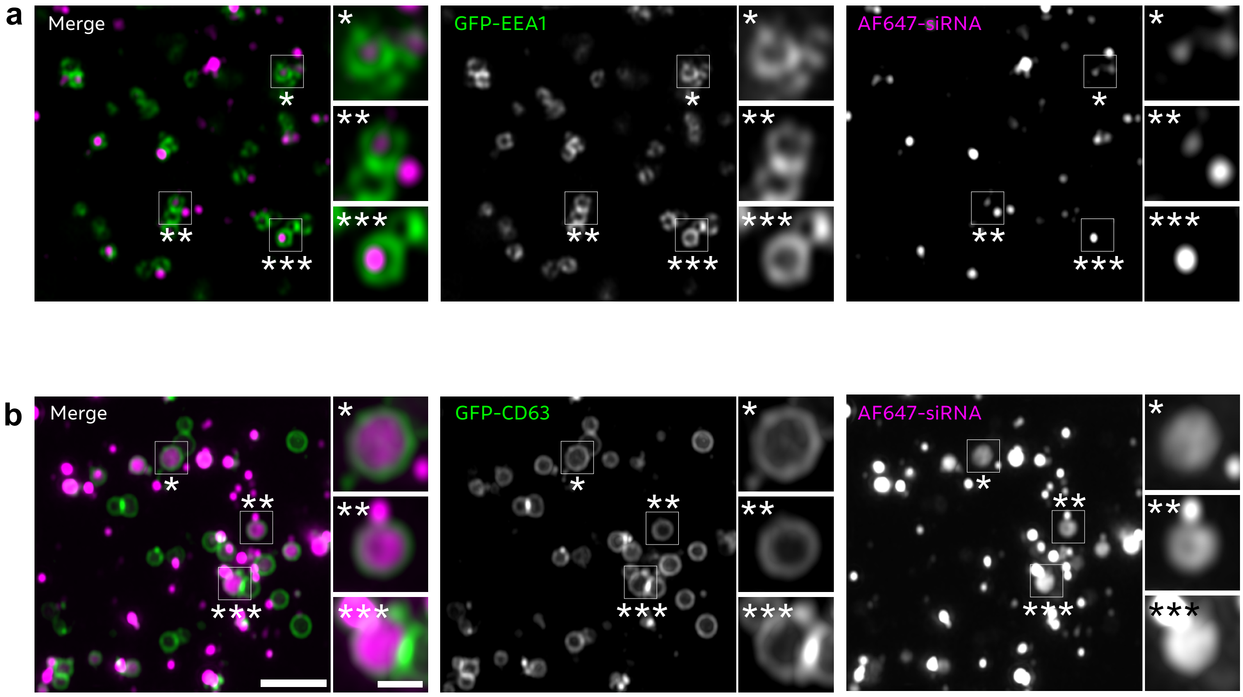

Supplementary Fig. 10 | Structure of disintegrating LNPs is preserved after PFA fixation. HeLa cells expressing GFP-EEA1 (**a**) or GFP-CD63 (**b**) were incubated with 100 nM AF647-siRNA-LNPs for 60 min (EEA1) or 120 min (CD63). Cells were fixed with PFA and imaged with an Airyscan confocal microscope. Brightness and contrast were adjusted separately for EEA1 and CD63 images. Scale bar, 2 µm; detail, 500 nm. Images are representative of 1 experiment.

Supplementary Note 1

Synthesis of BODIPY–MC3

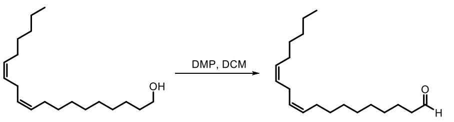

I. Dess-Martin periodinane (2.90 mL, 0.91 mmol) was added in one portion to a stirred suspension of (9Z,12Z)-octadeca-9,12-dien-1-ol (202.0 mg, 0.76 mmol) in DCM (5 mL) at 0°C. The resulting solution was allowed to come to room temperature over 4 hours. The reaction mixture was diluted with DCM (20 mL), and washed sequentially with saturated aqueous NaHCO3 (10 mL), and sat. Na2S2O3 (10 mL) The organic layer was dried over MgSO4, filtered and concentrated under reduced pressure to dryness to afford crude product. The resulting residue was purified by flash silica chromatography, elution gradient 0 to 30% EtOAc in hexanes. Product fractions were concentrated under reduced pressure to dryness to afford (9Z,12Z)-octadeca-9,12-dienal (120 mg, 59.7 %) as a colorless liquid. 1H NMR (500 MHz, Chloroform-d) δ ppm 0.9 - 0.9 (m, 3 H) 1.3 - 1.4 (m, 14 H) 1.6 - 1.7 (m, 2 H) 2.1 (q, J=6.8 Hz, 4 H) 2.4 - 2.5 (m, 2 H) 2.8 (t, J=6.6 Hz, 2 H) 5.3 - 5.4 (m, 4 H) 9.8 (s, 1 H).

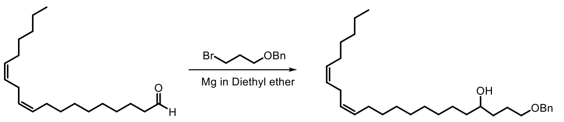

II. Iodine (12.00 mg, 0.05 mmol) was added in one portion to a stirred suspension of magnesium (207 mg, 8.51 mmol) and ((3-bromopropoxy)methyl)-benzene (0.501 mL, 2.84 mmol) in tetrahydrofuran (5 mL) at 25°C under argon. The reaction mixture was heated to 55°C over 30 min. At this point the color of the reaction mixture changes from orange to cloudy white. (9Z,12Z)-octadeca-9,12-dienal (250 mg, 0.95 mmol) was dissolved in 2 mL of THF and added dropwise to the solution. Color change from white/colorless to brown. Reaction mixture was then warmed up to 60 °C for 2 hours and allowed to come to RT overnight under Argon for 15 hours. The reaction mixture was cooled down and quenched with water (2 mL), followed by the addition of 1M HCl (10 mL) and DCM (15 mL). The layers were separated, and the aqueous layer was extracted with DCM (3 x 15 mL). The combined organic layers were washed with saturated aqueous NaCl (15 mL). The organic layer was dried over MgSO4, filtered and concentrated under reduced pressure to dryness to afford crude product. TLC shows formation of a less polar product (Rf = 0.6) in 4:1 hexanes/EtOAc. The resulting residue was purified by flash silica chromatography, elution gradient 0 to 55% hexanes in EtOAc. Product fractions were concentrated under reduced pressure to dryness to afford (12Z,15Z)-1-(benzyloxy)henicosa-12,15-dien-4-ol (268 mg, 68.3 %) as a colorless oil. 1H NMR (500MHz, Chloroform-d) 0.83 (t, J = 6.9 Hz, 3H), 1.21 - 1.44 (m, 19H), 1.54 - 1.71 (m, 3H), 1.93 - 2.03 (m, 4H), 2.03 - 2.12 (m, 1H), 2.71 (t, J = 6.7 Hz, 2H), 3.45 (t, J = 6.1 Hz, 2H), 3.50 - 3.57 (m, 1H), 4.46 (s, 2H), 5.24 - 5.35 (m, 4H), 7.19 - 7.30 (m, 5H); C28H46O2 m/z calcd. 414.674 observed 415.4 [M+H]+ (LCMS).

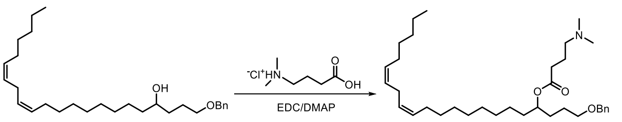

III. 3-(((ethylimino)methylene)amino)-N,N-dimethylpropan-1-amine hydrochloride (121 mg, 0.63 mmol) was added in one portion to a stirred solution of (12Z,15Z)-1-(benzyloxy)henicosa-12,15-dien-4-ol (124.7 mg, 0.30 mmol), 3-carboxy-N,N-dimethylpropan-1-aminium chloride (101 mg, 0.60 mmol), N,N-dimethylpyridin-4-amine (7.35 mg, 0.06 mmol) and N-ethyl-N-isopropylpropan-2-amine (0.220 mL, 1.26 mmol) in DCM (3 mL) at 0°C under argon. The resulting solution was stirred at 25 °C for 18 hours. The reaction mixture was diluted with DCM (10 mL) and water (10 mL). The layers were separated, and the aqueous layer was extracted with DCM (3 x 15 mL). The combined organic layers were washed with 0.5 M citric acid (10 mL). The organic layer was dried over MgSO4, filtered and concentrated under reduced pressure to dryness to afford crude product. The resulting residue was purified by flash silica chromatography, elution gradient 0 to 40% of [20% MeOH/DCM (w/1% NH4OH)] in DCM. Product fractions were concentrated under reduced pressure to dryness to afford (12Z,15Z)-1-(benzyloxy)henicosa-12,15-dien-4-yl 4-(dimethylamino)butanoate (124 mg, 78 %) as a yellow oil. 1H NMR (500MHz, Chloroform-d) 0.91 (t, J = 6.8 Hz, 3H), 1.30 (br s, 16H), 1.50 - 1.58 (m, 2H), 1.60 - 1.70 (m, 4H), 1.79 - 1.89 (m, 2H), 2.03 - 2.11 (m, 4H), 2.24 - 2.47 (m, 10H), 2.80 (t, J = 6.7 Hz, 2H), 3.48 (br s, 2H), 4.51 (s, 2H), 4.92 (br d, J = 5.3 Hz, 1H), 5.32 - 5.44 (m, 4H), 7.30 - 7.38 (m, 5H); C34H57NO3 m/z calcd. 527.434 observed 528.5 [M+H]+ (LCMS).

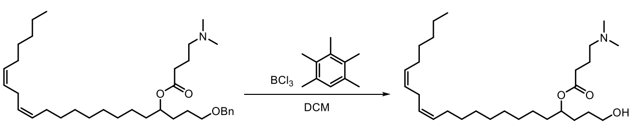

IV. Trichloroborane (0.248 mL, 0.25 mmol) was added dropwise to a stirred solution of (12Z,15Z)-1-(benzyloxy)henicosa-12,15-dien-4-yl 4-(dimethylamino)butanoate (65.4 mg, 0.12 mmol) and 1,2,3,4,5-pentamethylbenzene (55.1 mg, 0.37 mmol) in DCM (2.5 mL) at −78°C under argon. The resulting solution was allowed to stir at 0°C for 20 minutes. The reaction mixture was then cooled down to −78°C and quenched with 3 mL of MeOH:DCM (1:9) solution. Crude mixture was concentrated under reduced pressure to dryness. The resulting residue was purified by flash silica chromatography, elution gradient 0 to 50% of 20% MeOH/DCM (w/1% NH4OH) in DCM. Product fractions were concentrated under reduced pressure to dryness to afford (12Z,15Z)-1-hydroxyhenicosa-12,15-dien-4-yl 4-(dimethylamino)butanoate (17.70 mg, 32.6 %) as a colorless oil. 1H NMR (400 MHz, Methanol-d4) δ ppm 0.9 (t, J=6.7 Hz, 3 H) 1.3 (br d, J=2.9 Hz, 22 H) 1.8 (quin, J=7.5 Hz, 2 H) 2.0 - 2.1 (m, 4 H) 2.3 (s, 6 H) 2.3 - 2.4 (m, 4 H) 2.8 (t, J=6.1 Hz, 2 H) 3.5 (t, J=6.2 Hz, 2 H) 4.9 - 4.9 (m, 1 H) 5.3 - 5.4 (m, 4 H); C27H51NO3 m/z calcd. 437.387 observed 438.5 [M+H]+ (LCMS).

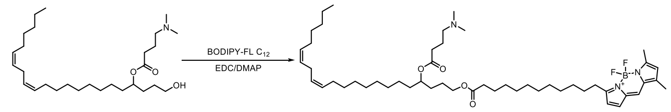

V. 3-(((ethylimino)methylene)amino)-N,N-dimethylpropan-1-amine hydrochloride (4.58 mg, 0.02 mmol) was added in one portion to a stirred solution of (12Z,15Z)-1-hydroxyhenicosa-12,15-dien-4-yl 4-(dimethylamino)-butanoate (7.85 mg, 0.02 mmol), 12-(5,5-difluoro-7,9-dimethyl-5H-4l4,5l4-dipyrrolo[1,2-c:2',1'-f][1,3,2]diazaborinin-3-yl)dodecanoic acid (5.00 mg, 0.01 mmol), N,N-dimethylpyridin-4-amine (0.292 mg, 2.39 µmol) and N-ethyl-N-isopropylpropan-2-amine (8.33 µl, 0.05 mmol) in DCM (2 mL) at 0°C under argon. The resulting solution was stirred at 25 °C for 18 hours. The reaction mixture was diluted with water (15 mL) and EtOAc (15 mL). The layers were separated, and the aqueous layer was extracted with EtOAc (3 x 15 mL). The combined organic layers was dried over MgSO4, filtered and concentrated under reduced pressure to dryness to afford crude product. The resulting residue was purified with prep SFC, on a Viridis BEH column, 250x30mm, 5 µm particle size, using a 20-22% gradient of EtOH/NH3 (20mM) in 7 minutes. The back pressure was set to 120 bar, the detection wavelength was 230 nm and the column temperature was 40 degrees C. To afford (12E,15E)-4-((4-(dimethylamino)butanoyl)oxy)henicosa-12,15-dien-1-yl 12-(5,5-difluoro-7,9-dimethyl-5H-4l4,5l4-dipyrrolo[1,2-c:2',1'-f][1,3,2]diazaborinin-3-yl)dodecanoate (8 mg, 39.9 %) as an oil. C50H82BF2N3O4 m/z calcd. 837.637 observed 838.6 [M+H]+ (LCMS).

!
