# Supplementary material for: Cellular and biophysical barriers to lipid nanoparticle mediated delivery of RNA to the cytosol
Source: Nat Commun. 2025 Jul 1;16:5354. doi: 10.1038/s41467-025-60959-z (PMC12217163; doi:10.1038/s41467-025-60959-z)
Supplement: Supplementary file 2 — Description of Additional Supplementary Files [file 41467_2025_60959_MOESM2_ESM.pdf]

Description of Additional Supplementary Files

File name: Supplementary Movie 1
Description: Galectin-9 recruitment to damaged vesicles during uptake of RNA-LNPs.
HeLa cells expressing YFP-Galectin-9 were treated with 50 nM AF647-siRNA-LNPs, 0.75 µg mL–1 Cy5-mRNA-LNPs or only imaging medium (control), and live-cell imaged every 10 min with a widefield microscope. Color bar indicate min and max intensity values. Scale bar is 50 µm. Time-lapse recording is representative of two independent experiments per RNA modality.

File name: Supplementary Movie 2
Description: siRNA-LNPs treatment induces recruitment of CHMP2A.
HeLa cells transiently expressing GFP-CHMP2A were incubated with 100 nM AF647-siRNA-LNPs and imaged every 10 min with widefield microscopy. Time-lapse recording is representative of 4 independent experiments.

File name: Supplementary Movie 3
Description: LNPs damage Rab5+EEA1+/- early endosomes.
HeLa cells expressing YFP-Galectin-9 and mScarlet-EEA1 or mScarlet-Rab5 were incubated with 50 nM AF647-siRNA-LNP and imaged with a high-speed widefield microscope. Circle indicate EEA1+/Rab5+ vesicle containing siRNA-LNP at t = 0, and pause indicate recruitment of galectin-9. Scale bar is 3 µm. Time-lapse recording is representative of 23 EEA1+ and 48 Rab5+ events from two independent experiments per compartment marker.

File name: Supplementary Movie 4
Description: 3D image sequence of intact or disintegrated LNPs in early or late endosomes.
HeLa cells expressing GFP-EEA1 or GFP-CD63 were incubated with 100 nM AF647-siRNA-LNPs for 30-90 min or 90-130 min for EEA1 or CD63 respectively. Cells were imaged live, as z-stacks with 21 slices and 100 nm step-size were obtained using VT-iSIM. z1 refers to the top plane and z21 to the bottom plane. Images are representative of 2 independent experiments. Scale bar is 500 nm.
