# Supplementary material for: Cellular and biophysical barriers to lipid nanoparticle mediated delivery of RNA to the cytosol
Source: Nat Commun. 2025 Jul 1;16:5354. doi: 10.1038/s41467-025-60959-z (PMC12217163; doi:10.1038/s41467-025-60959-z)
Supplement: Supplementary file 7 — Reporting Summary [file 41467_2025_60959_MOESM7_ESM.pdf]

nature portfolio

Corresponding author(s): Anders Wittrup

Last updated by author(s): May 15, 2025

Reporting Summary

Nature Portfolio wishes to improve the reproducibility of the work that we publish. This form provides structure for consistency and transparency in reporting. For further information on Nature Portfolio policies, see our Editorial Policies and the Editorial Policy Checklist.

Statistics

For all statistical analyses, confirm that the following items are present in the figure legend, table legend, main text, or Methods section.

n/a	Confirmed	
☐	☒	The exact sample size (*n*) for each experimental group/condition, given as a discrete number and unit of measurement
☐	☒	A statement on whether measurements were taken from distinct samples or whether the same sample was measured repeatedly
☐	☒	The statistical test(s) used AND whether they are one- or two-sided<br>*Only common tests should be described solely by name; describe more complex techniques in the Methods section.*
☒	☐	A description of all covariates tested
☐	☒	A description of any assumptions or corrections, such as tests of normality and adjustment for multiple comparisons
☐	☒	A full description of the statistical parameters including central tendency (e.g. means) or other basic estimates (e.g. regression coefficient) AND variation (e.g. standard deviation) or associated estimates of uncertainty (e.g. confidence intervals)
☐	☒	For null hypothesis testing, the test statistic (e.g. *F*, *t*, *r*) with confidence intervals, effect sizes, degrees of freedom and *P* value noted<br>*Give P values as exact values whenever suitable.*
☒	☐	For Bayesian analysis, information on the choice of priors and Markov chain Monte Carlo settings
☒	☐	For hierarchical and complex designs, identification of the appropriate level for tests and full reporting of outcomes
☒	☐	Estimates of effect sizes (e.g. Cohen's *d*, Pearson's *r*), indicating how they were calculated

Our web collection on statistics for biologists contains articles on many of the points above.

Software and code

Policy information about availability of computer code

Data collection	ZEN 2.3 (blue), ZEN 3.8.2 (black), ZEN 2.1 (black), VisiView Version 6.0.0.16, BD Accuri C6 Software Version 1.0.264.21, StepOne Software v2.3, FLUOstar OPTIMA Software v.1.32 R2
Data analysis	MATLAB R2019b, CellProfiler 2.1.1, GraphPad Prism 10 for macOS Version 10.0.2, Fiji 1.52i, Huygens Professional for Windows Version 17.04 and 23.04

For manuscripts utilizing custom algorithms or software that are central to the research but not yet described in published literature, software must be made available to editors and reviewers. We strongly encourage code deposition in a community repository (e.g. GitHub). See the Nature Portfolio guidelines for submitting code & software for further information.

Data

Policy information about availability of data

All manuscripts must include a data availability statement. This statement should provide the following information, where applicable:

- Accession codes, unique identifiers, or web links for publicly available datasets
- A description of any restrictions on data availability
- For clinical datasets or third party data, please ensure that the statement adheres to our policy

All source data underlying the quantitative figures are available in the source data file.
The MATLAB program for single vesicle tracking is available at https://github.com/hdurietz/QuantEscape. The MATLAB code used for analysis of sub-endosomal

localization of damage markers relative to LNP-siRNA, distance measurements of BODIPY-MC3+ and FRET+ foci from the nucleus, and colocalization analysis of RNA + endosomes with free BODIPY-MC3+ of dense BODIPY-MC3+ endosomes is available at https://github.com/hdurietz/BarriersToLNPdelivery.

Research involving human participants, their data, or biological material

Policy information about studies with human participants or human data. See also policy information about sex, gender (identity/presentation), and sexual orientation and race, ethnicity and racism.

Reporting on sex and gender	No research with human participants was performed in this manuscript.
Reporting on race, ethnicity, or other socially relevant groupings	*Please specify the socially constructed or socially relevant categorization variable(s) used in your manuscript and explain why they were used. Please note that such variables should not be used as proxies for other socially constructed/relevant variables (for example, race or ethnicity should not be used as a proxy for socioeconomic status).*<br>*Provide clear definitions of the relevant terms used, how they were provided (by the participants/respondents, the researchers, or third parties), and the method(s) used to classify people into the different categories (e.g. self-report, census or administrative data, social media data, etc.)*<br>*Please provide details about how you controlled for confounding variables in your analyses.*
Population characteristics	*Describe the covariate-relevant population characteristics of the human research participants (e.g. age, genotypic information, past and current diagnosis and treatment categories). If you filled out the behavioural & social sciences study design questions and have nothing to add here, write "See above."*
Recruitment	*Describe how participants were recruited. Outline any potential self-selection bias or other biases that may be present and how these are likely to impact results.*
Ethics oversight	*Identify the organization(s) that approved the study protocol.*

Note that full information on the approval of the study protocol must also be provided in the manuscript.

Field-specific reporting

Please select the one below that is the best fit for your research. If you are not sure, read the appropriate sections before making your selection.

[x] Life sciences [ ] Behavioural & social sciences [ ] Ecological, evolutionary & environmental sciences

For a reference copy of the document with all sections, see nature.com/documents/nr-reporting-summary-flat.pdf

Life sciences study design

All studies must disclose on these points even when the disclosure is negative.

Sample size	No predefined sample-size calculations were performed. Typically, as many data points (vesicles or cells) as possible were collected.
Data exclusions	Inclusion criteria for each specific quantification pipeline are described in detail in the Method section. Briefly, for single-veiscle tracking, non-trackable or quantifiable events were excluded. For manual classification of LNP integrity, a small subset of endosomes inspected were excluded where no obvious classification could be performed (generally because of too low RNA signal intensity). For determination of sub-endosomal localization of LNPs and damage markers in single vesicles, non-quantifiable clustered endosomes and endosomes with centralized localization of the LNP or damage marker within the endosome were excluded.
Replication	Most experiments were replicated in at least two independent experiments, with the exception of LC-MS of BODIPY-MC3 LNPs, where three biological samples (per treatment) were used, and evaluation of how PFA fixation affected LNP integrity, where a large number of cells and individual endosomes where evaluated. Also, disintegration assessment using full-cell images was performed ones, since nearly identical experiments with higher magnification images were repeated at least three times for all conditions, with consistent results.
Randomization	Samples were not randomized. Only in vitro experiments were performed, where randomization is not practical. Furthermore, typically unbiased algorithm-based quantifications were performed, eliminating the need for sample randomization.
Blinding	Generally, no blinding strategy was needed, since observer independent algorithms were mainly used for quantifications. For calculating de novo CHMP2A foci recruitment following LNP treatment, the individual counting the CHMP2A foci was blinded to treatment allocation.

Reporting for specific materials, systems and methods

We require information from authors about some types of materials, experimental systems and methods used in many studies. Here, indicate whether each material, system or method listed is relevant to your study. If you are not sure if a list item applies to your research, read the appropriate section before selecting a response.

Materials & experimental systems

n/a	Involved in the study	
☒	☐	Antibodies
☐	☒	Eukaryotic cell lines
☒	☐	Palaeontology and archaeology
☒	☐	Animals and other organisms
☒	☐	Clinical data
☒	☐	Dual use research of concern
☒	☐	Plants

Methods

n/a	Involved in the study	
☒	☐	ChIP-seq
☐	☒	Flow cytometry
☒	☐	MRI-based neuroimaging

Eukaryotic cell lines

Policy information about cell lines and Sex and Gender in Research

Cell line source(s)	HeLa cells purchased from American Type Culture Collection (ATCC).
Authentication	Genotyping according to ANSI/ATCC standard ASN-0002 (Eurofins) with STR and by continuous cell morphology and growth monitoring.
Mycoplasma contamination	Cells were regularly tested and confirmed negative for Mycoplasma infection.
Commonly misidentified lines (See ICLAC register)	Not included.

Plants

Seed stocks	*Report on the source of all seed stocks or other plant material used. If applicable, state the seed stock centre and catalogue number. If plant specimens were collected from the field, describe the collection location, date and sampling procedures.*
Novel plant genotypes	*Describe the methods by which all novel plant genotypes were produced. This includes those generated by transgenic approaches, gene editing, chemical/radiation-based mutagenesis and hybridization. For transgenic lines, describe the transformation method, the number of independent lines analyzed and the generation upon which experiments were performed. For gene-edited lines, describe the editor used, the endogenous sequence targeted for editing, the targeting guide RNA sequence (if applicable) and how the editor was applied.*
Authentication	*Describe any authentication procedures for each seed stock used or novel genotype generated. Describe any experiments used to assess the effect of a mutation and, where applicable, how potential secondary effects (e.g. second site T-DNA insertions, mosiacism, off-target gene editing) were examined.*

Flow Cytometry

Plots

Confirm that:

☐ The axis labels state the marker and fluorochrome used (e.g. CD4-FITC).

☐ The axis scales are clearly visible. Include numbers along axes only for bottom left plot of group (a 'group' is an analysis of identical markers).

☐ All plots are contour plots with outliers or pseudocolor plots.

☐ A numerical value for number of cells or percentage (with statistics) is provided.

Methodology

Sample preparation	Details are provided in the Method section. Briefly, cultured HeLa cells were washed with PBS and detached through trypsinization. The samples were then washed with, and ultimately resuspended in, 0.5% Bovine Serum Albumin in PBS.
Instrument	BD Accuri C6 Cytometer. Becton Dickinson, Franklin Lakes, NJ, USA.
Software	BD Accuri C6 Software Version 1.0.264.21.
Cell population abundance	N/A. Single population in homogenous cell line was used.
Gating strategy	Viable cells were gated based on FSC/SSC scatter.

☐ Tick this box to confirm that a figure exemplifying the gating strategy is provided in the Supplementary Information.
